# RAVINDER LAL KAPUR (1938–2006)

**Published:** 2006

**Authors:** Ajit V. Bhide

**Affiliations:** * Head Department of Psychiatry, St Martha's Hospital, Bangalore; Associate, National Institute of Advanced Studies, Bangalore; *mailing address:* Vasant Vihar, 79, Amarjyoti Layout, Sanjay Nagar, Bangalore 560094, Karnataka; e-mail: avb3004@hotmail.com


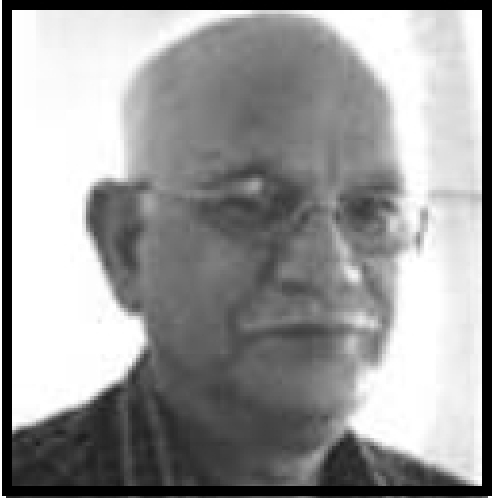


Untimely and sudden death snatched from our midst a great teacher, thinker and psychiatrist. Professor R.L. Kapur died in Bellagio, Italy, while on a writing sabbatical, on Friday, 24 November 2006. His beloved wife Professor Malavika Kapur was with him at the time of his demise from a myocardial infarction.

Born in pre-partition West Punjab in 1938, Ravinder Lal Kapur had his medical education at Amritsar. He qualified with a DPM from the All India Institute of Mental Health, Bangalore (the present NIMHANS) receiving a Gold Medal. He then won a Commonwealth scholarship for higher studies—the first psychiatrist to do so—which took him to Edinburgh where he earned his PhD, his guide being Professor Norman Kreitman.

After working for several years in the UK, Professor Kapur returned to India and joined the Kasturba Medical College, Manipal. There he was influential in upgrading the Department of Psychiatry. He then joined NIMHANS as professor of Community Psychiatry. Epidemiological work carried out during his days at Manipal led to the publication of the first Indian monograph on psychiatric epidemiology, *The Great Universe of Kota* (with Professor G.M. Carstairs, Hogarth Press, 1976). In the same year, he became head of the Department of Psychiatry at NIMHANS and was, till then, the youngest person to ever head a postgraduate department of psychiatry in the country. He continued at NIMHANS till 1983. Then followed a stint at the Centre for Theoretical Studies at the Indian Institute of Science (IISc), Bangalore and his entry into private practice, which he carried on with till the very end.

He joined the National Institute of Advanced Studies (NIAS), at the IISc campus in Bangalore, when it was founded in 1988, and was later its Deputy Director. At the time of his passing away, he was Emeritus Professor there. Colleagues and students at NIAS admired his great skills as both an analyser of data and a synthesizer of knowledge. Professor Kapur had also held visiting professorships at various institutions including Harvard University. As a specialist consultant to World Health Organization (WHO), he worked in Somalia during troubled times.

Impatient with the mundane, he chose unusual areas to explore. Among these we could include work to study the psyche of terrorists in Punjab and Kashmir, the alienation of youth in late twentieth-century India, studies of senior administrative officers' job orientation and satisfaction from a psychological perspective, creativity among Indian scientists and his research on spirituality. He was the first and possibly the only psychiatrist to have taken a sabbatical to study the subjective experiences of yogic practices, under the aegis of the Indian Council of Medical Research (ICMR). He inter-viewed the Dalai Lama with great savvy for an international news channel, and later held a no-holds-barred interview with the redoubtable K.P.S. Gill who is believed to have been instrumental in containing terrorism in the early 1990s. All these pursuits have sometimes earned him the sobriquet of being a ‘fringe psychiatrist’; an unfair sobriquet, when one recalls that he had never forsaken hard-core psychiatry, was always happy to be back in his clinical practice and relished clinical discussions.

A recipient of numerous prestigious awards and fellow-ships, Dr Kapur was well known for his sharp intellect and the talent to distil and condense knowledge. This made him a teacher with a remarkable ability to present even very convoluted concepts in a simple fashion. A warm-hearted human being, he endeared himself effortlessly to almost all who came in touch with him. An erudite speaker and engaging participant in academic meets, Dr Kapur had inimitable spontaneity and wit. During his days as professor at NIMHANS, an academic atmosphere was created that encouraged intellectual debate, gave rise to phenomenal teaching programmes that have stood the test of time, and everyone from the junior-most students to the senior-most faculty enjoyed full freedom of expression.

Many revolutionary changes were made at NIMHANS during his time, and his keen interest in psychosocial aspects of psychiatry was wrongly interpreted as being in opposition to the swelling tide in favour of biological psychiatry. He was, in fact, instrumental in setting up the now well-known department of psychopharmacology at NIMHANS. He weathered many a storm in those days, never relinquishing his stance supporting a multidisciplinary approach, and minimizing role hierarchy.

All those who worked in his unit remember the challenging yet friendly atmosphere he helped create. Many of his students who are now heads of units and departments, acknowledge his being their role model. As a thesis guide, examiner and chairman of academic sessions, he was known to be stimulating though unsparing in his criticism, which never failed to be constructive. He was always delighted with his students' achievements but lamented at times that some did not keep in touch with him. Dr Kapur espoused the cause of ethical practice with passion.

Though he listed psychiatric epidemiology, cross-cultural psychiatry, psychotherapy and yoga as his main areas of interest, Dr Kapur was keenly interested in every aspect of mental health, and was a self-avowed perpetual learner. He was on the first editorial board of *Culture, Medicine and Psychiatry* and had served in editorial capacity of several national and international publications. His constant effort as a writer and editor was to simplify language while retaining the general effect. He was famously exasperated by the bombastic and the inelegant.

For well over a decade he was pursuing his interest in studying spirituality, and was researching the lives of rishis and sadhus in the Himalayas, and expanding on the mental health implications of his findings. He undertook several Himalayan sojourns to catch up on his subjects, and even in his sixties, seemed youthful and rejuvenated after each mountain trip. His interest in spirituality never distanced him from his abiding faith in rationality. At Bellagio, he was on assignment writing about his research with rishis and sadhus. Less than a month earlier, he had delivered a path-breaking guest lecture on this very topic, at the Annual Conference of the IPS–South Zone, at Mangalore.

Dr Kapur was a great lover of music, and he learnt Hindustani vocal for several years. A gracious host and remarkable conversationalist, he had the ability to ‘walk with kings, not lose the common touch’. Dr Kapur used to list Sri Aurobindo, the late Professor N.C. Surya and Professor J.S. Neki as important influences in his life. He told me that he had learnt a great deal also from Professor Neki's poetry in his practice of healing and his outlook on life.

The very first recipient of the Marfatia Award (1966), he also received the Bhagwat Award (with Dr R. Raguram, 1983), and the late Dr D.L.N. Murthy Rao Memorial Oration Award (1994), of the IPS. The Karnataka Chapter of IPS had also bestowed on him its Eminent Psychiatrist Award (1993) and the Dr Achar Memorial Oration Award (1998). He was also the Chairman of the ANCIPS Organizing Committee in 1996. The IPS will miss his friendly and wise guidance and his august presence.

Professor Kapur is survived by his wife, Dr Malavika (retired professor of clinical psychology); daughter, Dr Svapna Sabnis (paediatrician); and son, Dr Sharad Kapur, and three grandchildren. Even as we join his family in mourning the passing away of this most uncommon soul, we celebrate the chance for having known him.

